# Real-World Evidence Study on the Long-Term Safety of Everolimus in Patients With Tuberous Sclerosis Complex: Final Analysis Results

**DOI:** 10.3389/fphar.2022.802334

**Published:** 2022-04-08

**Authors:** María Luz Ruiz-Falcó Rojas, Martha Feucht, Alfons Macaya, Bernd Wilken, Andreas Hahn, Ricardo Maamari, Yulia Hirschberg, Antonia Ridolfi, John Chris Kingswood

**Affiliations:** ^1^ Hospital Infantil Universitario Nino Jesus, Madrid, Spain; ^2^ Universitäts-Klinik für Kinder-und Jugendheilkunde Wien, Vienna, Austria; ^3^ Hospital Universitari Vall d’ Hebron, Barcelona, Spain; ^4^ Klinikum Kassel GmbH, Kassel, Germany; ^5^ Abteilung Kinderneurologie, Universitätsklinikum Giessen und Marburg GmbH, Giessen, Germany; ^6^ Novartis Pharmaceuticals Corporation, East Hanover, NJ, United States; ^7^ Novartis Pharma S.A.S, Rueil-Malmaison, France; ^8^ Royal Sussex County Hospital, Brighton, United Kingdom

**Keywords:** tuberous sclerosis complex (TSC), everolimus, TOSCA, post-authorization safety study (PASS), real-world evidence (RWE)

## Abstract

The TuberOus SClerosis registry to increase disease Awareness (TOSCA) Post-Authorization Safety Study (PASS) was a non-interventional, multicenter, safety substudy that assessed the long-term safety of everolimus in patients with tuberous sclerosis complex (TSC) receiving everolimus for its licensed indications in the European Union (EU). This substudy also aimed to address TSC-associated neuropsychiatric disorders (TAND), sexual development, and male infertility. Eligible patients were enrolled from 39 sites across 11 countries in the EU. Outcomes of interest included the incidence of adverse events (AEs), serious adverse events (SAEs), treatment-related AEs (TRAEs), AEs leading to everolimus discontinuation, AEs of special interest (AESIs), the observed relationship between everolimus blood levels and incidence of AESIs, TAND, and reproductive clinical features. Herein, we present the final analysis results from this substudy (data cutoff date: 22 January 2020). At data cutoff, 179 patients were enrolled (female, 59.2%; age ≥18 years, 65.9%), of which the majority completed the study (76%). Overall, 121 patients (67.6%) had AEs regardless of causality. The most frequent TRAEs (≥5%) were stomatitis (7.8%), aphthous ulcer (6.7%), and hypercholesterolemia (6.1%). The most common treatment-related SAEs (>1%) were pneumonia (3.4%), influenza, pyelonephritis, aphthous ulcer, stomatitis, dyslipidemia, and hypercholesterolemia (1.1% each). Ten patients (5.6%) reported AEs leading to everolimus discontinuation. The common psychiatric disorders (*N* = 179) were autism spectrum disorder (21.8%), anxiety disorder (12.8%), “other” psychiatric disorders (8.9%), attention-deficit hyperactivity disorder, and depressive disorder (7.8% each). Of 179 patients, 88 (49.2%) had ≥1 behavioral problem. Of these (*n* = 88), the most common (>20%) were sleep difficulties (47.7%), anxiety (43.2%), mood swings (37.5%), depression mood (35.2%), impulsivity (30.7%), severe aggression (23.9%), and overactivity (22.7%). Of 179 patients, four (2.2%) reported abnormal puberty onset, and three (1.7%) reported other reproductive disorders. Of 106 females, 23 (21.7%) reported menstrual cycle disorders and 10 (9.4%) reported amenorrhea. Available data did not show delays in sexual maturation or an association between sexual development and infertility. The results demonstrate that everolimus has a manageable long-term safety profile in the TSC treatment setting. No new safety signals emerged. This substudy also contributed to the mapping of TAND and reproductive clinical features in patients with TSC.

## Introduction

Tuberous sclerosis complex (TSC) is a rare, multisystem, autosomal dominant disorder characterized by the growth of benign tumors (hamartomas) in various organs, including the brain, kidneys, lungs, liver, heart, and skin (Crino et al., 2006; Curatolo et al., 2008). TSC is caused by inactivating mutations in the *TSC1* or *TSC2* genes (Dabora et al., 2001; Crino et al., 2006; Curatolo et al., 2008; Huang and Manning, 2008; Tyburczy et al., 2015). These mutations lead to a hyperactivation of the mammalian target of rapamycin (mTOR) pathway with subsequent abnormal cell proliferation/differentiation, which in turn results in the development of hamartomatous lesions characteristic of TSC (Zhang et al., 2003; Kwiatkowski and Manning, 2005; Jozwiak et al., 2008; Borkowska et al., 2011).

Patients with TSC experience a wide spectrum of clinical manifestations with varying degrees of severity as well as age-related onset and expression patterns (Józwiak et al., 2000; Crino et al., 2006; Curatolo et al., 2008; Franz et al., 2010; Kingswood et al., 2014; Curatolo et al., 2015). Central nervous system (CNS) manifestations, such as cortical tubers, subependymal nodules, and subependymal giant cell astrocytoma (SEGA), are common in TSC and cause significant disease burden (Curatolo et al., 2008; Franz et al., 2010; Curatolo and Maria, 2013; Curatolo et al., 2015). The majority of patients develop epilepsy that is often refractory (Franz et al., 2010; Kingswood et al., 2014). In addition, TSC-associated neuropsychiatric disorders (TAND) significantly impact quality of life (Krueger and Northrup, 2013; Curatolo et al., 2015); TAND features comprise a wide range of manifestations, such as intellectual disability, academic/scholastic difficulties, autism spectrum disorders and other neurodevelopmental and psychiatric disorders, and various behavioral problems (Prather and de Vries, 2004; Krueger and Northrup, 2013; de Vries et al., 2015).

Targeting the mTOR pathway has emerged as a promising therapeutic strategy for TSC (Davies et al., 2011; McCormack et al., 2011; Krueger and Northrup, 2013; Tran and Zupanc, 2015; Capal and Franz, 2016; Franz and Krueger, 2018; Annear et al., 2019). Everolimus is a selective and orally bioavailable mTOR inhibitor approved for the treatment of adult and pediatric patients aged ≥1 year who have TSC-associated SEGA that requires therapeutic intervention but is not amenable to surgery. Everolimus is also approved for the treatment of adults with TSC-related renal angiomyolipoma not requiring immediate surgery. Since 2017, everolimus has been approved as an adjunctive treatment for patients with TSC aged ≥2 years who have refractory partial-onset seizures, with or without secondary generalization (US Food and Drug Administration, 2018; European Medicines Agency, 2020). The approvals were based on the results from the EXIST-1 (Franz et al., 2013; Franz et al., 2014; Franz et al., 2016), EXIST 2 (Bissler et al., 2013; Bissler et al., 2016; Bissler et al., 2017), and EXIST 3 (French et al., 2016; Franz et al., 2018) phase 3, randomized, double-blind, placebo-controlled clinical trials, which demonstrated the efficacy and safety of everolimus in these indications. Furthermore, results from the long-term follow-up of patients in these randomized clinical trials showed that everolimus had a manageable long-term safety and tolerability profile. Stomatitis was the most frequently reported treatment-related adverse event (TRAE) (Franz et al., 2016; Bissler et al., 2017; Franz et al., 2018).

There is limited real-world evidence of the long-term safety of everolimus for its licensed indications in the TSC treatment setting. The TuberOus SClerosis registry to increase disease Awareness (TOSCA) was a global registry primarily established to address gaps in understanding the course of various TSC manifestations, therapeutic interventions and their outcomes, and quality of life (Kingswood et al., 2014; Jansen et al., 2020). The registry aimed at collecting data from patients with TSC to further inform treatment standards and promote research in TSC (Kingswood et al., 2014; Kingswood et al., 2017).

The TOSCA Post-Authorization Safety Study (PASS) was a non-interventional, multicenter, safety substudy that was initiated based on a request from the European Medicines Agency (EMA) (Kingswood et al., 2014). The purpose of this safety substudy was to prospectively collect data on the long-term safety profile of everolimus prescribed for TSC-related licensed indications in a real-world setting in the European Union (EU) (Kingswood et al., 2014). The TOSCA PASS also aimed to address TAND, sexual development, and male infertility.

Results of a prior interim analysis from TOSCA PASS (data cutoff: 10 August 2017) showed that everolimus had a manageable safety profile in patients with TSC who received everolimus for the licensed indications (Kingswood et al., 2021). Furthermore, data on reproductive clinical features indicated age-appropriate sexual maturation in these patients (Kingswood et al., 2021).

Herein, we present final analysis results from TOSCA PASS that include cumulative data from the PASS first patient first visit (FPFV, 7 March 2013) to the last date of study data collection (data cutoff date: 22 January 2020). The focus of this report will be on the long-term safety of everolimus and TAND features.

## Materials and Methods

### Study Design and Participants

The TOSCA PASS (EU PAS Register Number ENCePP/SDPP/3247) enrolled patients with TSC from 39 sites across 11 countries in the EU. The participating countries were Austria, Czech Republic, Denmark, France, Germany, Netherlands, Poland, Slovenia, Spain, Sweden, and the United Kingdom. Patients receiving everolimus prescribed for its licensed indications were eligible. No clinical, instrumental, or laboratory assessments/interventions were performed in this study other than those required for disease management, according to local best practice, or required to monitor any treatment as per locally approved summary of product characteristics. Due to the observational nature of this study, no specific visit schedules were mandated, and only available data from routine clinical management of patients were collected at patients’ visits to their site. Patients could be withdrawn from the study if any of the following occurred: death, lost to follow-up by the site, voluntary withdrawal of consent, or at physician discretion. The period of observation in this study ended before the start of the COVID-19 pandemic in Europe.

This study was designed, implemented, and reported in accordance with the Guidelines for Good Pharmacoepidemiology Practices (GPP) of the International Society for Pharmacoepidemiology (ISPE 2008), the Strengthening the Reporting of Observational Studies in Epidemiology (STROBE) guidelines, and the ethical principles in the Declaration of Helsinki. The protocol and the proposed informed consent form were reviewed and approved by constituted Institutional Review Boards/Independent Ethics Committees/Research Ethics Boards (IRB/IEC/REB). Patients (or parent/guardian as applicable) had to sign the TOSCA PASS informed consent form before any data or information were provided for this study.

### TOSCA PASS Objectives and Endpoints

The main objective of the TOSCA PASS was to document the long-term safety and tolerability profile of everolimus prescribed for the licensed indications in patients with TSC residing in the EU (Kingswood et al., 2014). The corresponding endpoints included the incidence of adverse events (AEs), serious adverse events (SAEs), TRAEs, AEs leading to everolimus discontinuation, and the incidence of AEs of special interest (AESIs) (Kingswood et al., 2014); AESIs were those events of special clinical interest that were explored in relation to everolimus treatment (Supplementary Table S2), and these events have also been listed in the prior interim analysis report (Kingswood et al., 2021). The other objective was to collect everolimus Therapeutic Drug Monitoring data, and the corresponding endpoint was everolimus blood concentration, where available (Kingswood et al., 2014). The relationship among everolimus blood levels, the incidence of AESIs, and the intake of concomitant antiepileptic drugs (AEDs) was also explored. In addition, the TOSCA PASS aimed to address TAND, sexual development, and male infertility.

### Data Collection

The start of data collection was 7 March 2013 (PASS FPFV). The recommended frequency of data collection was 3-monthly intervals, with a minimum of once-yearly follow-up visits (10 ± 2 months interval) for each patient to ensure an ongoing data stream. Disease evaluation could be performed more frequently, if needed. For reporting purposes, the baseline visit defined each subsequent follow-up window (FU1, FU2, FU3, FU4, and FU5, etc). All events occurring during the 12 months on or after the baseline visit were reported as baseline; all events in the next 12-month period were reported as follow-up 1, and so forth. This was derived for each patient regardless of the actual dates of the visits. A follow-up observation period of up to five years was foreseen for PASS patients aged >16 years for females or >17 years for males, or at Tanner stage V. Follow-up visits were scheduled according to the standard practice of each site and as per the treating physician’s judgment. For pediatric patients, the follow-up period was extended until they reached Tanner stage V, or until age 16 for females or 17 for males, regardless of the end of treatment, to collect long-term data on safety, sexual maturation, and fertility. Patients who chose to withdraw consent were not contacted for follow-up information.

An interim analysis from this study was submitted to the EMA every year. In agreement with the EMA, the TOSCA PASS was terminated early since no new safety signals were identified, and due to the observational nature of the study, further data collection was not expected to provide meaningful data to draw new conclusions.

### Statistical Analysis

Variables of interest were summarized descriptively either for the overall population and by age ranges or at baseline and by follow-up year. In this manuscript, data by follow-up included only those reported for follow-up year 1–6, while the data from follow-up year 7 were not reported, owing to very few patients. AEs were assessed according to the Common Terminology Criteria for Adverse Events (CTCAE) version 4.03. The incidence of various AEs was summarized by preferred terms using the Medical Dictionary for Regulatory Activities (MedDRA) version 22.1.

## Results

### Patient Disposition and Baseline Characteristics

**FIGURE 1:**
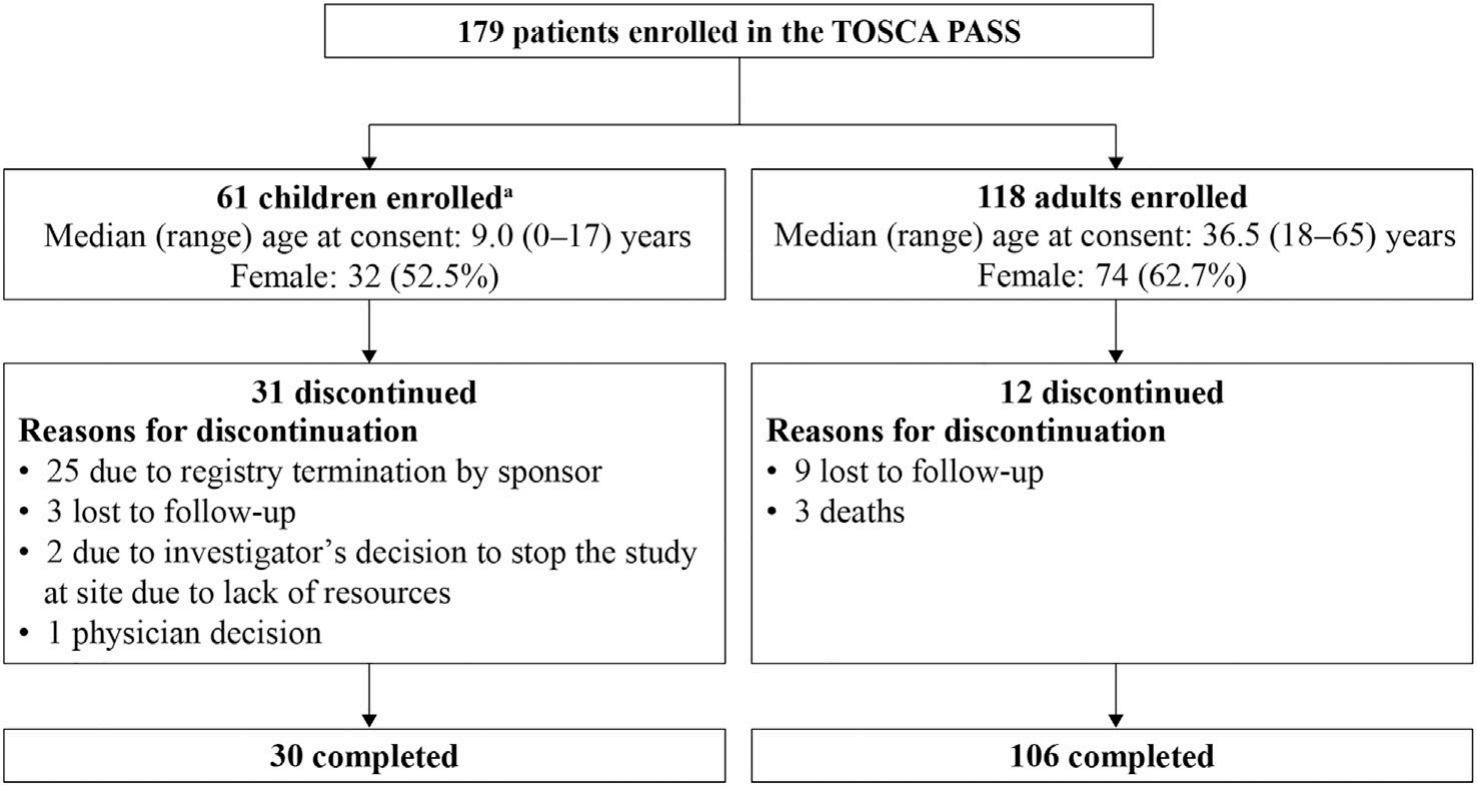
Patient disposition in the TOSCA PASS.PASS, Post-Authorization Safety Study.^a^Children within different age groups at registry entry were as follows: ≤2 years (*n* = 7), >2 to ≤9 years (*n* = 27), and >9 to <18 years (*n* = 27).

Overall, 179 patients from 11 European countries participating in the TOSCA registry were enrolled in the TOSCA PASS. Of these, the majority (*n* = 136, 76%) completed the study, while 43 (24%) discontinued. The primary reasons for discontinuation were registry termination by the sponsor (*n* = 25, 14%), loss to follow-up (*n* = 12, 6.7%), death (*n* = 3, 1.7%), investigator’s decision to stop the study at site due to lack of resources (*n* = 2, 1.1%), and physician’s decision (*n* = 1, 0.6%). The patient disposition is presented in Figure 1.

Of 179 enrolled patients, 106 (59.2%) were female. The mean age at consent was 27.1 years, and the median age was 27.0 years (range, 0–65 years). Patients within different age groups at registry entry were as follows: ≤2 years (*n* = 7); >2 to ≤9 years (*n* = 27); >9 to <18 years (*n* = 27); and ≥18 years (*n* = 118). The proportion of adults (≥18 years) was numerically higher than the proportion of pediatric patients (*n* = 118, 65.9% vs. *n* = 61, 34.1%). Detailed disease characteristics of the enrolled patients have been previously published (Kingswood et al., 2021). Of the 179 patients enrolled, 100 (55.9%) had SEGA, 149 (83.2%) had renal angiomyolipoma, and 151 (84.4%) had epilepsy (Kingswood et al., 2021).

### Everolimus Dosage, Exposure, and Safety

Table 1 presents everolimus dosage, exposure, and safety in the overall population (*N* = 179), by sex, and across age groups. Most patients took the tablet formulation (*n* = 172, 96.1%). The most commonly administered dosage was 5 mg (*n* = 156, 87.2%). The average (standard deviation [SD]) daily dose was 7.3 mg (3.1 mg), and the median duration of exposure was 1026.0 days (range, 36–2652 days). Everolimus dose changes were reported in 116 patients (64.8%); dose interruptions in 69 (38.5%), dose increases in 98 (54.7%), and dose reductions in 57 (31.8%) patients. In 78 patients (43.6%), the reason for dose changes was listed as “other”, with no additional specific details. In 50 patients (27.9%), the reason for dose changes was side effects.

**TABLE 1:** Everolimus dosage, exposure, and safety for the overall population, by sex, and across age groups.

Category	Overall	By sex		By age at consent, years			
	*N* = 179 (100%)	Female *N* = 106	Male *N* = 73	≤2 *N* = 7 (100%)	>2 to ≤9 *N* = 27 (100%)	>9 to <18 *N* = 27 (100%)	≥18 *N* = 118 (100%)
Pharmaceutical formulation^a^, *n* (%)							
Tablets	172 (96.1)	101 (95.3)	71 (97.3)	6 (85.7)	25 (92.6)	23 (85.2)	118 (100.0)
Dispersible tablets	13 (7.3)	8 (7.5)	5 (6.8)	3 (42.9)	5 (18.5)	5 (18.5)	0
Dosage^a^, *n* (%)							
2 mg	3 (1.7)	2 (1.9)	1 (1.4)	1 (14.3)	0	0	2 (1.7)
2.5 mg	28 (15.6)	19 (17.9)	9 (12.3)	3 (42.9)	8 (29.6)	3 (11.1)	14 (11.9)
3 mg	5 (2.8)	4 (3.8)	1 (1.4)	2 (28.6)	2 (7.4)	1 (3.7)	0
5 mg	156 (87.2)	92 (86.8)	64 (87.7)	5 (71.4)	20 (74.1)	22 (81.5)	109 (92.4)
10 mg	28 (15.6)	16 (15.1)	12 (16.4)	1 (14.3)	6 (22.2)	9 (33.3)	12 (10.2)
Other	54 (30.2)	33 (31.1)	21 (28.8)	4 (57.1)	14 (51.9)	13 (48.1)	23 (19.5)
Daily dose, mg							
Average (SD)	7.3 (3.1)	7.4 (3.5)	7.2 (2.6)	4.1 (2.0)	6.2 (2.4)	7.9 (2.3)	7.7 (3.3)
Median (min–max)	7.3 (1–20)	7.3 (1–20)	7.3 (3–15)	4.5 (1–7)	5.6 (3–10)	7.5 (4–13)	7.4 (1–20)
Patients with dose changes^a^, *n* (%)	116 (64.8)	68 (64.2)	48 (65.8)	7 (100.0)	19 (70.4)	19 (70.4)	71 (60.2)
Interruptions	69 (38.5)	39 (36.8)	30 (41.1)	5 (71.4)	12 (44.4)	8 (29.6)	44 (37.3)
Increases	98 (54.7)	57 (53.8)	41 (56.2)	7 (100.0)	18 (66.7)	17 (63.0)	56 (47.5)
Reductions	57 (31.8)	34 (32.1)	23 (31.5)	3 (42.9)	11 (40.7)	13 (48.1)	30 (25.4)
Reasons for changes^a^, *n* (%)							
Side effect	50 (27.9)	30 (28.3)	20 (27.4)	4 (57.1)	9 (33.3)	8 (29.6)	29 (24.6)
Dosing error	5 (2.8)	3 (2.8)	2 (2.7)	1 (14.3)	0	1 (3.7)	3 (2.5)
Lab test abnormality	7 (3.9)	4 (3.8)	3 (4.1)	0	1 (3.7)	4 (14.8)	2 (1.7)
Concomitant medication affecting drug exposure	4 (2.2)	3 (2.8)	1 (1.4)	2 (28.6)	1 (3.7)	0	1 (0.8)
Other	78 (43.6)	43 (40.6)	35 (47.9)	7 (100.0)	18 (66.7)	12 (44.4)	41 (34.7)
Duration of exposure^b^, days							
Mean (SD)	1146.4 (503.9)	1113.7 (468.1)	1194.0 (551.7)	1482.6 (971.7)	1728.4 (599.2)	1200.6 (422.0)	981.0 (324.6)
Median (min–max)	1026.0 (36–2652)	1033.5 (83–2368)	1016.0 (36–2652)	1824.0 (83–2368)	1919.0 (216–2652)	1141.0 (490–2135)	982.0 (36–1743)
Overall blood levels of everolimus, ng/ml							
Mean (SD)	5.5 (4.0)	5.7 (4.4)	5.2 (3.3)	4.5 (2.2)	5.7 (3.4)	6.8 (2.9)	5.2 (4.4)
Median (min–max)	4.5 (1.0–35.9)	4.4 (1.0–35.9)	4.7 (1.3–20.7)	3.9 (1.9–8.2)	4.7 (1.0–13.3)	6.4 (2.5–12.3)	4.2 (1.2–35.9)
Blood levels of everolimus by number of AEs, type of AEs, and concomitant AEDs, ng/ml							
No AE	73	40	33	1	5	12	55
Mean (SD)	5.5 (4.9)	5.7 (5.8)	5.4 (3.5)	3.9 (-)	4.8 (1.9)	6.6 (2.7)	5.4 (5.5)
Median (min–max)	4.3 (1.3–35.9)	4.1 (1.6–35.9)	4.8 (1.3–20.7)	3.9 (3.9–3.9)	5.0 (2.0–7.0)	6.7 (2.6–10.6)	4.1 (1.3–35.9)
≥1 AE	102	65	37	6	21	14	61
Mean (SD)	5.6 (3.3)	5.9 (3.4)	4.9 (2.9)	4.6 (2.4)	6.3 (3.6)	7.4 (3.3)	5.0 (3.1)
Median (min–max)	4.7 (1.0–15.2)	4.9 (1.0–15.2)	4.3 (1.0–13.0)	4.6 (1.9–8.2)	5.0 (1.0–12.3)	6.1 (2.5–12.3)	4.3 (1.0–15.2)
≥1 grade 3/4 AE	25	17	8	3	6	4	12
Mean (SD)	4.3 (1.9)	4.3 (1.9)	4.2 (2.0)	3.0 (0.4)	4.4 (2.3)	5.8 (0.6)	4.0 (2.0)
Median (min–max)	4.2 (1.4–8.3)	4.8 (1.4–8.3)	3.2 (2.1–7.1)	3.2 (2.5–3.2)	4.0 (2.0–7.1)	5.9 (5.3–6.3)	4.1 (1.4–8.3)
≥1 AESI	73	47	26	5	17	12	39
Mean (SD)	6.4 (4.5)	6.6 (3.4)	6.2 (6.2)	5.0 (2.4)	7.7 (4.4)	9.0 (7.1)	5.3 (3.4)
Median (min–max)	5.1 (1.0–29.0)	5.6 (1.2–15.2)	4.3 (1.0–29.0)	5.6 (1.9–8.2)	6.2 (1.8–18.3)	6.5 (2.5–29.0)	4.3 (1.0–15.2)
≥1 grade 3/4 AESI	16	10	6	2	3	3	8
Mean (SD)	4.3 (2.0)	4.3 (2.2)	4.3 (1.8)	3.6 (0.5)	4.0 (2.7)	5.7 (0.6)	4.0 (2.3)
Median (min–max)	4.1 (1.4–8.3)	4.5 (1.4–8.3)	3.6 (2.7–7.1)	3.6 (3.2–3.9)	3.0 (2.0–7.1)	5.4 (5.3–6.3)	3.5 (1.4–8.3)
≥1 concomitant AED	67	39	28	6	20	14	27
Mean (SD)	6.5 (5.1)	7.2 (6.0)	5.5 (3.2)	3.9 (1.6)	6.0 (3.5)	7.9 (3.0)	6.7 (7.0)
Median (min–max)	5.0 (1.0–35.9)	5.1 (1.0–35.9)	4.9 (1.3–15.9)	3.7 (1.9–5.9)	5.0 (1.0–13.3)	8.4 (3.0–12.3)	4.5 (1.2–35.9)
≥1 AESI and ≥1 concomitant AED	38	23	15	4	12	6	16
Mean (SD)	6.3 (5.1)	6.9 (3.8)	5.3 (6.7)	4.2 (1.9)	6.7 (3.6)	11.7 (8.9)	4.4 (3.4)
Median (min–max)	5.1 (1.2–29.0)	5.6 (1.2–15.2)	4.0 (1.3–29.0)	4.6 (1.9–5.9)	5.3 (1.8–12.0)	8.8 (5.3–29.0)	4.1 (1.2–15.2)

AE, adverse event; AED, antiepileptic drug; AESI, adverse event of special interest; SD, standard deviation.

a A patient may have taken both formulations, multiple dosages, or may have had multiple reasons for dose changes.

b Duration of exposure was “end date − start date + 1”.

Only data with collection date before or on study completion date were analyzed.

The overall mean (SD) blood level of everolimus (*n* = 175) was 5.5 ng/ml (4.0 ng/ml). The mean (SD) blood levels of everolimus (in ng/ml) in patients who experienced no AE, ≥1 AE, ≥1 grade 3/4 AE, ≥1 AESI, and ≥1 grade 3/4 AESI were 5.5 (4.9), 5.6 (3.3), 4.3 (1.9), 6.4 (4.5), and 4.3 (2.0), respectively. In patients who had ≥1 AESI and were treated with ≥1 concomitant AED, the mean (SD) blood level of everolimus was 6.3 ng/ml (5.1 ng/ml) (Table 1).

There were no differences in everolimus dosing or blood levels between males and females. Everolimus blood levels were not different between age groups, but there was a trend to an increased mean/median dose in adults vs. children (Table 1).

### Safety

#### Concomitant Medications

Of 179 patients, 120 (67.0%) received concomitant medications or significant non-drug therapies. The most frequently administered concomitant medications were nervous system medications (*n* = 95, 53.1%) including vigabatrin (*n* = 32, 17.9%), lamotrigine (*n* = 29, 16.2%), levetiracetam (*n* = 25, 14.0%), valproate sodium (*n* = 20, 11.2%), and oxcarbazepine (*n* = 19, 10.6%); medications belonging to the alimentary tract and metabolism class (*n* = 60, 33.5%) including colecalciferol (*n* = 25, 14.0%); and systemic antiinfectives (*n* = 38, 21.2%) including cefuroxime (*n* = 10, 5.6%). Sirolimus (0.1% and 0.25%) was used topically for angiofibroma in one patient (not related to AE), and sirolimus (6-mg dose) was also used for anemia in another patient (not related to AE).

#### Adverse Events

Overall, 121 patients (67.6%) had AEs regardless of causality; 44 patients (24.6%) had grade 1, 32 (17.9%) had grade 2, 36 (20.1%) had grade 3, and nine (5.0%) had grade 4 AEs. Supplementary Table S1 presents frequent AEs (>3% overall) regardless of causality. The most frequent AE regardless of causality was stomatitis (*n* = 16, 8.9%).

Overall, AEs suspected to be everolimus-related (TRAEs) were reported in 81 patients (45.3%). The rate of TRAEs was numerically higher in children vs. adults (n/N = 42/61, 68.9% vs. n/N = 39/118, 33.1%). Frequent TRAEs (>3% overall) were reported in 49 patients (grade 1 [*n* = 27, 15.1%], grade 2 [*n* = 15, 8.4%], grade 3 [*n* = 7, 3.9%]) (Table 2). The most frequent (>5%) were stomatitis (*n* = 14, 7.8%), aphthous ulcer (*n* = 12, 6.7%), and hypercholesterolemia (*n* = 11, 6.1%) (Table 2). The rates of frequent TRAEs were comparable between females and males (Table 2).

**TABLE 2:** Frequent TRAEs (>3%, overall) in the overall population, by sex, and across age groups.

Category	Overall	By sex		By age at consent, years			
	*N* = 179	Female *N* = 106	Male *N* = 73	≤2 *N* = 7	>2 to ≤9 *N* = 27	>9 to <18 *N* = 27	≥18 *N* = 118
Patients with any frequent AEs suspected to be everolimus-related, *n* (%)	49 (27.4)	30 (28.3)	19 (26.0)	5 (71.4)	12 (44.4)	11 (40.7)	21 (17.8)
Stomatitis	14 (7.8)	7 (6.6)	7 (9.6)	2 (28.6)	4 (14.8)	5 (18.5)	3 (2.5)
Aphthous ulcer	12 (6.7)	7 (6.6)	5 (6.8)	1 (14.3)	3 (11.1)	1 (3.7)	7 (5.9)
Hypercholesterolemia	11 (6.1)	8 (7.5)	3 (4.1)	1 (14.3)	4 (14.8)	3 (11.1)	3 (2.5)
Pneumonia	8 (4.5)	3 (2.8)	5 (6.8)	2 (28.6)	3 (11.1)	0	3 (2.5)
Headache	7 (3.9)	5 (4.7)	2 (2.7)	0	0	2 (7.4)	5 (4.2)
Hypertriglyceridemia	6 (3.4)	5 (4.7)	1 (1.4)	0	0	4 (14.8)	2 (1.7)
Mouth ulceration	6 (3.4)	5 (4.7)	1 (1.4)	1 (14.3)	2 (7.4)	2 (7.4)	1 (0.8)
Patients with frequent AEs suspected to be everolimus-related with CTC grade^a^, *n* (%)	49 (27.4)	30 (28.3)	19 (26.0)	5 (71.4)	12 (44.4)	11 (40.7)	21 (17.8)
Grade 1	27 (15.1)	17 (16.0)	10 (13.7)	2 (28.6)	8 (29.6)	7 (25.9)	10 (8.5)
Grade 2	15 (8.4)	9 (8.5)	6 (8.2)	2 (28.6)	1 (3.7)	2 (7.4)	10 (8.5)
Grade 3	7 (3.9)	4 (3.8)	3 (4.1)	1 (14.3)	3 (11.1)	2 (7.4)	1 (0.8)

AEs, adverse events; CTC, Common Terminology Criteria; CTCAE, Common Terminology Criteria for Adverse Events; MedDRA, Medical Dictionary for Regulatory Activities; TRAEs, treatment-related adverse events.

MedDRA version 22.1 and CTCAE version 4.03 were used.

a If a patient reported multiple frequent AEs, the frequent AE with the worst severity was used.

SAEs regardless of causality were reported in 59 patients (33.0%); 58 (32.4%) had SAEs with Common Terminology Criteria (CTC) grade reported (grade 1 [*n* = 7, 3.9%], grade 2 [*n* = 11, 6.1%], grade 3 [*n* = 31, 17.3%], grade 4 [*n* = 9, 5.0%]). The most frequent SAEs regardless of causality (>3% overall) were pneumonia (*n* = 8, 4.5%) and epilepsy (*n* = 7, 3.9%).

SAEs suspected to be everolimus-related were reported in 25 patients (14%); four patients (2.2%) had grade 1, six (3.4%) had grade 2, 13 (7.3%) had grade 3, and two (1.1%) had grade 4 SAEs. The most common SAEs (>1% overall) suspected to be everolimus-related were pneumonia (*n* = 6, 3.4%), influenza, pyelonephritis, aphthous ulcer, stomatitis, dyslipidemia, and hypercholesterolemia (*n* = 2, 1.1% each).

AEs leading to everolimus discontinuation were reported in 10 patients (5.6%); four patients (2.2%) had grade 1, and two (1.1%) each had grade 2, grade 3, and grade 4 AEs. The AEs leading to study drug discontinuation were fatigue, amenorrhea (*n* = 2, 1.1% each), anemia, mouth ulceration, drug ineffective, empyema, pneumonia, hyperglycemia, type I diabetes mellitus, flank pain, intestinal adenocarcinoma, seizure, and alopecia (*n* = 1, 0.6% each). AEs leading to everolimus dose adjustments were reported in 60 patients (grade 1 [*n* = 23, 12.8%], grade 2 [*n* = 17, 9.5%], grade 3 [*n* = 19, 10.6%], grade 4 [*n* = 1, 0.6%]). The most frequent (>2% overall) were diarrhea, aphthous ulcer (*n* = 6, 3.4% each), pneumonia (*n* = 5, 2.8%), stomatitis, nasopharyngitis, and urinary tract infection (*n* = 4, 2.2% each).

Overall, AESIs were reported in 91 patients (50.8%). The most frequent AESI by safety topic of interest was severe infections (*n* = 64, 35.8%); the events occurring in >2% of patients were nasopharyngitis (*n* = 11, 6.1%), pneumonia, urinary tract infection (*n* = 10, 5.6% each), bronchitis (*n* = 7, 3.9%), influenza (*n* = 6, 3.4%), ear infection, gastroenteritis, oral candidiasis, pharyngitis, and rhinitis (*n* = 4, 2.2% each) (Supplementary Table S2). Increased creatinine or proteinuria or renal failure was observed in three patients (1.7%); the events included increased blood creatinine (*n* = 1, 0.6%) and proteinuria (*n* = 3, 1.7%).

With longer follow-up in this study, the number of deaths remained unchanged relative to that reported in the prior interim analysis (Kingswood et al., 2021). A total of three deaths were reported, and none were related to everolimus treatment. Detailed narratives for these deaths have been published previously (Kingswood et al., 2021).

#### Everolimus Blood Levels and AESIs

Of 179 TOSCA PASS patients, information related to everolimus exposure was available for 150 patients at the baseline visit, while 29 entered the PASS after the baseline visit. Of these 150 patients, the majority did not experience AESIs (*n* = 101, 67.3%); 49 (32.7%) experienced AESIs, and in 37 (24.7%), these AESIs were suspected to be everolimus-related. Similar trends were observed at follow-up 1–4 (Table 3).

**TABLE 3:** AESIs and incidence by everolimus blood levels and number of concomitant AEDs at baseline and by follow-up year.

Category	Baseline *N* = 150	FU1 *N* = 171	FU2 *N* = 164	FU3 *N* = 112	FU4 *N* = 71	FU5 *N* = 22	FU6 *N* = 8
Patients on treatment with everolimus, *n* (%)							
No AESI	101 (67.3)	114 (66.7)	116 (70.7)	77 (68.8)	50 (70.4)	8 (36.4)	4 (50.0)
AESI present	49 (32.7)	57 (33.3)	48 (29.3)	35 (31.3)	21 (29.6)	14 (63.6)	4 (50.0)
AESI suspected to be everolimus-related	37 (24.7)	38 (22.2)	35 (21.3)	20 (17.9)	11 (15.5)	8 (36.4)	2 (25.0)
Patients who experienced AESI during everolimus treatment by everolimus blood level, *n* (%)							
<5 ng/ml	22 (14.7)	28 (16.4)	22 (13.4)	12 (10.7)	5 (7.0)	3 (13.6)	2 (25.0)
5–15 ng/ml	23 (15.3)	25 (14.6)	16 (9.8)	14 (12.5)	12 (16.9)	5 (22.7)	2 (25.0)
>15 ng/ml	1 (0.7)	3 (1.8)	3 (1.8)	3 (2.7)	2 (2.8)	0	2 (25.0)
Patients who experienced AESI during everolimus treatment by number of concomitant AEDs, *n* (%)							
With concomitant^a^ use of any AEDs	24 (16.0)	25 (14.6)	23 (14.0)	20 (17.9)	13 (18.3)	11 (50.0)	4 (50.0)
With concomitant use of ≥2 AEDs	16 (10.7)	16 (9.4)	16 (9.8)	8 (7.1)	8 (11.3)	8 (36.4)	3 (37.5)
With concomitant use of ≥3 AEDs	6 (4.0)	9 (5.3)	8 (4.9)	3 (2.7)	5 (7.0)	5 (22.7)	2 (25.0)
With concomitant use of ≥4 AEDs	2 (1.3)	3 (1.8)	3 (1.8)	1 (0.9)	2 (2.8)	3 (13.6)	1 (12.5)

AEDs, antiepileptic drugs; AESIs, adverse events of special interest; FU, follow-up; FUP, follow-up period.

a Concomitant use of AED was defined as the use of AED taken between the start date and the stop date of AESI.

An event was mapped into baseline/FUP k if its start date was prior to the baseline date/baseline date + 12 × k months and its stop date was on or after the baseline date/baseline date + 12 × k months or the event was ongoing, where k = 1, 2, 3, 4, 5, 6.

Among patients who experienced AESIs during everolimus treatment, 22 (14.7%) had everolimus blood levels <5 ng/ml, 23 (15.3%) had everolimus blood levels in the range of 5–15 ng/ml, and one (0.7%) had everolimus blood levels >15 ng/ml. Similar proportions were observed in follow-up 1–4 (Table 3).

The proportion of patients who experienced AESIs during everolimus treatment and with concomitant AED use at baseline and follow-up 1–6 is shown in Table 3.

### TSC-Associated Neuropsychiatric Disorders (TAND)

Overall, investigator-reported data for TAND features were scarce compared to those for other TSC manifestations. In general, TAND features presented at varying frequencies across different age groups (Table 4 **)**.

**TABLE 4:** TAND by age group.

Category	Overall	By age at consent, years			
	*N* = 179	≤2 *N* = 7	>2 to ≤9 *N* = 27	>9 to <18 *N* = 27	≥18 *N* = 118
Common behavioral problems in TSC					
Patients with at least one behavioral problem^a^	88 (49.2)	6 (85.7)	21 (77.8)	19 (70.4)	42 (35.6)
Sleep difficulties					
Yes	42 (47.7)	4 (66.7)	14 (66.7)	9 (47.4)	15 (35.7)
No	32 (36.4)	2 (33.3)	6 (28.6)	8 (42.1)	16 (38.1)
Unknown	14 (15.9)	0	1 (4.8)	2 (10.5)	11 (26.2)
Severe aggression					
Yes	21 (23.9)	3 (50.0)	5 (23.8)	6 (31.6)	7 (16.7)
No	52 (59.1)	3 (50.0)	15 (71.4)	13 (68.4)	21 (50.0)
Unknown	15 (17.0)	0	1 (4.8)	0	14 (33.3)
Self-injury					
Yes	15 (17.0)	1 (16.7)	11 (52.4)	1 (5.3)	2 (4.8)
No	56 (63.6)	5 (83.3)	10 (47.6)	17 (89.5)	24 (57.1)
Unknown	17 (19.3)	0	0	1 (5.3)	16 (38.1)
Impulsivity					
Yes	27 (30.7)	3 (50.0)	8 (38.1)	8 (42.1)	8 (19.0)
No	44 (50.0)	3 (50.0)	12 (57.1)	11 (57.9)	18 (42.9)
Unknown	17 (19.3)	0	1 (4.8)	0	16 (38.1)
Overactivity					
Yes	20 (22.7)	3 (50.0)	7 (33.3)	4 (21.1)	6 (14.3)
No	51 (58.0)	3 (50.0)	14 (66.7)	14 (73.7)	20 (47.6)
Unknown	17 (19.3)	0	0	1 (5.3)	16 (38.1)
Depression mood					
Yes	31 (35.2)	0	4 (19.0)	6 (31.6)	21 (50.0)
No	46 (52.3)	6 (100.0)	16 (76.2)	12 (63.2)	12 (28.6)
Unknown	11 (12.5)	0	1 (4.8)	1 (5.3)	9 (21.4)
Anxiety					
Yes	38 (43.2)	0	10 (47.6)	7 (36.8)	21 (50.0)
No	38 (43.2)	6 (100.0)	11 (52.4)	11 (57.9)	10 (23.8)
Unknown	12 (13.6)	0	0	1 (5.3)	11 (26.2)
Mood swings					
Yes	33 (37.5)	1 (16.7)	10 (47.6)	6 (31.6)	16 (38.1)
No	40 (45.5)	5 (83.3)	10 (47.6)	12 (63.2)	13 (31.0)
Unknown	15 (17.0)	0	1 (4.8)	1 (5.3)	13 (31.0)
Obsession					
Yes	14 (15.9)	0	4 (19.0)	3 (15.8)	7 (16.7)
No	56 (63.6)	6 (100.0)	15 (71.4)	15 (78.9)	20 (47.6)
Unknown	18 (20.5)	0	2 (9.5)	1 (5.3)	15 (35.7)
Hallucination					
Yes	4 (4.5)	0	0	0	4 (9.5)
No	66 (75.0)	5 (83.3)	20 (95.2)	18 (94.7)	23 (54.8)
Unknown	18 (20.5)	1 (16.7)	1 (4.8)	1 (5.3)	15 (35.7)
Psychosis					
Yes	8 (9.1)	1 (16.7)	4 (19.0)	0	3 (7.1)
No	61 (69.3)	4 (66.7)	16 (76.2)	18 (94.7)	23 (54.8)
Unknown	19 (21.6)	1 (16.7)	1 (4.8)	1 (5.3)	16 (38.1)
Psychiatric disorders					
ASD					
Yes	39 (21.8)	2 (28.6)	15 (55.6)	7 (25.9)	15 (12.7)
No	63 (35.2)	4 (57.1)	11 (40.7)	18 (66.7)	30 (25.4)
Not done	77 (43.0)	1 (14.3)	1 (3.7)	2 (7.4)	73 (61.9)
ADHD					
Yes	14 (7.8)	1 (14.3)	8 (29.6)	1 (3.7)	4 (3.4)
No	78 (43.6)	5 (71.4)	16 (59.3)	21 (77.8)	36 (30.5)
Not done	87 (48.6)	1 (14.3)	3 (11.1)	5 (18.5)	78 (66.1)
Depressive disorder					
Yes	14 (7.8)	0	1 (3.7)	2 (7.4)	11 (9.3)
No	81 (45.3)	6 (85.7)	23 (85.2)	20 (74.1)	32 (27.1)
Not done	84 (46.9)	1 (14.3)	3 (11.1)	5 (18.5)	75 (63.6)
Anxiety disorder					
Yes	23 (12.8)	0	6 (22.2)	4 (14.8)	13 (11.0)
No	70 (39.1)	6 (85.7)	18 (66.7)	18 (66.7)	28 (23.7)
Not done	86 (48.0)	1 (14.3)	3 (11.1)	5 (18.5)	77 (65.3)
Other psychiatric disorder					
Yes	16 (8.9)	0	4 (14.8)	4 (14.8)	8 (6.8)
No	79 (44.1)	6 (85.7)	20 (74.1)	19 (70.4)	34 (28.8)
Not done	84 (46.9)	1 (14.3)	3 (11.1)	4 (14.8)	76 (64.4)
Intellectual ability					
Intellectual ability measured					
Yes	59 (33.0)	5 (71.4)	23 (85.2)	15 (55.6)	16 (13.6)
No	42 (23.5)	2 (28.6)	3 (11.1)	11 (40.7)	26 (22.0)
Unknown	78 (43.6)	0	1 (3.7)	1 (3.7)	76 (64.4)
Number of patients with IQ score^a^	57 (31.8)	4 (57.1)	22 (81.5)	15 (55.6)	16 (13.6)
Normal	16 (28.1)	1 (25.0)	6 (27.3)	3 (20.0)	6 (37.5)
Mild intellectual disability	23 (40.4)	1 (25.0)	9 (40.9)	7 (46.7)	6 (37.5)
Moderate intellectual disability	13 (22.8)	1 (25.0)	5 (22.7)	3 (20.0)	4 (25.0)
Severe intellectual disability	10 (17.5)	2 (50.0)	2 (9.1)	3 (20.0)	3 (18.8)
Profound intellectual disability	3 (5.3)	0	3 (13.6)	0	0
Neuropsychological skills assessment					
Yes^a^	61 (34.1)	7 (100.0)	21 (77.8)	14 (51.9)	19 (16.1)
No	32 (17.9)	0	5 (18.5)	10 (37.0)	17 (14.4)
Not done	86 (48.0)	0	1 (3.7)	3 (11.1)	82 (69.5)
Patients with any deficit^b^	47 (77.0)	6 (85.7)	15 (71.4)	12 (85.7)	14 (73.7)
Academic/scholastic skills difficulties					
Yes^a^	74 (41.3)	3 (42.9)	21 (77.8)	23 (85.2)	27 (22.9)
No	19 (10.6)	1 (14.3)	4 (14.8)	3 (11.1)	11 (9.3)
Not done	86 (48.0)	3 (42.9)	2 (7.4)	1 (3.7)	80 (67.8)
Patients with assessed difficulties	34 (45.9)	1 (33.3)	12 (57.1)	11 (47.8)	10 (37.0)

ADHD, attention-deficit hyperactivity disorder; ASD, autism spectrum disorder; IQ, intelligence quotient; TAND, TSC-associated neuropsychiatric disorders; TSC, tuberous sclerosis complex.

a Used as denominator to calculate percent rates for each subcategory.

b Performance <5th percentile.

Data are represented as n (%).

At the psychiatric level, the common psychiatric disorders reported (Table 4) were autism spectrum disorder (n/N = 39/179, 21.8%), anxiety disorder (n/N = 23/179, 12.8%), “other” psychiatric disorders (n/N = 16/179, 8.9%), attention-deficit hyperactivity disorder, and depressive disorder (n/N = 14/179, 7.8% each).

At the behavioral level, of 179 patients, 88 (49.2%) had at least one behavioral problem (Table 4). Of these, the most common (reported in >20%) were sleep difficulties (n/N = 42/88, 47.7%), anxiety (n/N = 38/88, 43.2%), mood swings (n/N = 33/88, 37.5%), depression mood (n/N = 31/88, 35.2%), impulsivity (n/N = 27/88, 30.7%), severe aggression (n/N = 21/88, 23.9%), and overactivity (n/N = 20/88, 22.7%).

At the intellectual level, an intelligence quotient (IQ) score was available for 57 of 179 patients (31.8%). Of these, 16/57 (28.1%) had normal intellectual ability, while mild, moderate, severe, and profound intellectual disabilities were observed in 23/57 (40.4%), 13/57 (22.8%), 10/57 (17.5%), and 3/57 (5.3%), respectively, (Table 4).

At the academic/scholastic level, 74 of 179 patients (41.3%) reported having had difficulties in school subjects, of which 34/74 (45.9%) had assessed difficulties. At the neuropsychological level, neuropsychological skills were formally assessed in 61 of 179 patients (34.1%). Of those assessed, neuropsychological deficits (performance <5th percentile) were identified in 47/61 (77%) (Table 4).

### Reproductive Clinical Features

Reproductive clinical features for the overall population and by age group are presented in Supplementary Table S3. Of 179 patients, four (2.2%) reported abnormal puberty onset (one male and three females). Menstrual cycle disorders (n/N = 23/106, 21.7%) and amenorrhea (n/N = 10/106, 9.4%) were reported in a low percentage of females aged ≥10 years. Other abnormal reproductive conditions were reported in three of 179 patients (1.7%).

Tanner staging was performed in 34 of 179 patients (19.0%; six males and 28 females). Male patients had genitalia stages 3 (n/N = 1/6, 16.7%), 4 (n/N = 3/6, 50.0%), and 5 (n/N = 2/6, 33.3%) and pubic hair stages 4 and 5 (n/N = 3/6, 50% each). Female patients mostly had breast stage 5 (n/N = 16/25, 64.0%) and pubic hair stage 5 (n/N = 18/26, 69.2%).

Of 106 females, a small proportion used contraception (*n* = 19, 17.9%), mostly hormone-based contraception (n/N = 16/19, 84.2%). Of 179 patients, five (2.8%) used external sex hormones and three (1.7%) underwent ovariectomy.

Overall, 39 of 179 patients (21.8%) underwent hormone tests (Supplementary Table S3); hormone tests were performed for 26 patients (14.6%) at baseline (*N* = 178), 19 (10.7%) at follow-up 1 (*N* = 178), 15 (8.5%) at follow-up 2 (*N* = 176), 11 (6.8%) at follow-up 3 (*N* = 161), two (2.2%) at follow-up 4 (*N* = 89), two (3.3%) at follow-up 5 (*N* = 61), and for none of the patients at follow-up 6 (*N* = 16).

## Discussion

The results of this final analysis from the TOSCA PASS demonstrated that everolimus had a manageable long-term safety and tolerability profile in patients with TSC. The safety profile of everolimus in this study was largely consistent with that previously reported in the TSC treatment setting (Krueger et al., 2013; Franz et al., 2014; Bissler et al., 2016; Franz et al., 2016; Bissler et al., 2017). Overall, AEs regardless of causality were reported in about two-thirds of patients, with most AEs of modest severity. The events were treatable with dose adjustments and/or use of concomitant medications. AEs leading to everolimus discontinuation were reported in a low percentage of patients in this study (5.6%), which was numerically lower compared with the rates of discontinuation due to AEs observed in the EXIST-1 (n/N = 11/111; 9.9%), EXIST-2 (n/N = 10/112; 8.9%), and EXIST-3 trials (n/N = 47/361; 13%) reporting on the long-term safety of everolimus (Franz et al., 2016; Bissler et al., 2017; Franz et al., 2018). One possible explanation for this is that clinicians in real life practice routinely commence everolimus at a lower dose than that used initially in the registration trials (Iqbal et al., 2017). The original starting dose chosen for everolimus in the EXIST-2 study (10 mg/day) was based on the fact that 10 mg was found to be tolerated by most adults in oncology trials (Davies et al., 2017). During the EXIST-2 study, it was found that 71.4% of patients had dose interruptions/reductions and after one year on everolimus, 35% received everolimus 5 mg/day. However, continued therapeutic benefits were still observed (Bissler et al., 2016; Davies et al., 2017; Northrup et al., 2021). Over the next five years in clinical practice, it was found that starting with 5 mg caused less side effects, was clinically effective, and very few patients needed a higher dose (Davies et al., 2017). A starting dose of 5 mg daily of everolimus in adults has become the almost universal practice of all experienced prescribers, as evidenced by the data in TOSCA.

In this substudy, numerically higher rates of TRAEs were observed in children vs. adults. We could speculate that some children may have needed a lower dose of everolimus than the one recommended, and thus, a higher exposure could have been achieved. Another explanation could be that pediatric patients may have been more thoroughly monitored for side effects compared with adult patients or may have had more frequent visits to the doctors’ offices and were therefore more closely followed up by their treating physicians. Nevertheless, due to the observational nature of this study, we cannot rule out the underreporting of AEs in the adult population. The most likely reason for this is that TOSCA is a non-interventional study, and as such, the reporting of AEs was done as per real-world practice, which might differ from randomized controlled studies (Franz et al., 2016; French et al., 2016; Bissler et al., 2017).

The results from this PASS also indicate that there was no cumulative toxicity (or new safety issues) observed with prolonged use of everolimus. Consistent with the long-term safety reports from the EXIST 1-3 clinical trials (Franz et al., 2016; Bissler et al., 2017; Franz et al., 2018), stomatitis was the most commonly reported (7.8%) TRAE in this substudy. Stomatitis-related AEs are a known identified risk associated with everolimus in patients with TSC, which are usually effectively managed to minimize their occurrence and severity (Kingswood et al., 2021). Severe infections, an identified risk with everolimus (Krueger et al., 2013; Trelinska et al., 2015; Bissler et al., 2017), were reported in 35.8% of patients in this substudy, mostly nasopharyngitis followed by pneumonia, urinary tract infection, bronchitis, and influenza. In the EXIST-2 trial report of the long-term safety of everolimus following four years of follow-up, infections were reported in 91.1% of patients, mostly those of the upper respiratory tract (Bissler et al., 2017). In this substudy, no conclusions could be drawn regarding the observed relationship between everolimus blood levels and AESI. The average blood levels of everolimus were not different in patients who did or who did not have AEs.

Consistent with prior TAND findings from the TOSCA study (Kingswood et al., 2017; de Vries et al., 2018), the results of this analysis from TOSCA PASS showed low reporting rates of TAND evaluation and reporting in patients with TSC in the EU, thus emphasizing the need for a more cautious evaluation of TAND by clinicians. The high rates of missing data limit the interpretability of the results of TAND features and, overall, are suggestive of underdiagnosis/undertreatment of TAND features in the clinical setting. In general, low rates of psychiatric disorders and behavioral difficulties were observed in this substudy. This finding is largely in line with the previously reported TAND findings from the TOSCA study (Kingswood et al., 2017; de Vries et al., 2018). At the neuropsychological level, of those who had neuropsychological skills assessed, 77% (n/N = 47/61) reported performance <5th percentile in this substudy compared with 55.7% (n/N = 314/564) in a prior TAND report from TOSCA (de Vries et al., 2018). At the academic level, of those who reported difficulties in school subjects or academic performance, the rates of individuals with assessed difficulties was 45.9% (n/N = 34/74) in this substudy vs. 48.8% (n/N = 359/735) in the prior TAND report from TOSCA (de Vries et al., 2018). In this study, 31.8% of patients had IQ scores, and among these, the rates of severe or profound intellectual disability were low.

With longer follow-up in this study, the rates of various reproductive clinical features remained either unchanged or numerically similar to those reported in the prior interim analysis (Kingswood et al., 2021). In general, limited data were collected for sexual maturation and hormone levels for patients in TOSCA PASS. Although the number of patients with reproductive hormonal testing performed decreased through the study, precluding a proper longitudinal evaluation and conclusion, overall, a low incidence of abnormal puberty onset, other abnormal reproductive conditions, amenorrhea, and menstrual cycle disorders was observed in this study. Overall, the available data did not suggest any delay in sexual maturation. A relationship between sexual development and male infertility could not be established.

Some limitations of the TOSCA PASS warrant discussion. Firstly, given the observational nature of the study, an important limitation was the high rates of missing data due to different reasons such as data not being reported, unknown data, or data not assessed by investigators. Of note, the missing data for the assessment and reporting of TAND likely reflect the limited use of this evaluation in the medical community. Secondly, the limited data on reproductive clinical features, in particular Tanner staging, possibly reflect gaps in the current medical practice for the reporting and/or assessment of reproductive clinical features in most European countries. Tanner staging is not routinely done as a standard practice as reproductive clinical features are not always considered a priority by the treating physicians, since patients with TSC experience multiple comorbidities. Moreover, Tanner stage evaluation assesses physical measurements of development based on external primary and secondary sex characteristics. Although an individual can reach the final Tanner stage (mature), this might not necessarily predict a final outcome on male or female infertility. Any impact on fertility would be observed when an individual tries to generate an offspring. Finally, considering the disease complexity, a patient was not always followed for all disease manifestations over follow-up periods by the site(s). Given the observational nature of the study, only data already available from clinical practice were collected.

In conclusion, TOSCA PASS provided a detailed picture of the TSC population in Europe. Results from this substudy show that everolimus has a well-characterized and acceptable long-term safety profile for its licensed indications in patients with TSC. No new safety signals were identified. AEs were common, but rarely needed withdrawal. This implies that everolimus treatment was considered valuable enough to continue despite AEs, and physicians therefore managed these events with a combination of dose interruptions and/or dose changes. The results from this study also suggest that physicians judge that the TSC indication is controlled on fairly low blood levels of everolimus. The observed occurrence of AEs did not seem to be influenced by everolimus blood levels. Although monitoring of renal function was not systematic, there were no strong indicators of any renal function-related issues in patients who were monitored. The results from this PASS showed inadequate monitoring and/or reporting of TAND in patients with TSC in the EU. Therefore, the effect of everolimus on TAND could not be interpreted from the collected data. However, despite some unavailable data, differences in local clinical practice, and the inconsistent application of international guidelines for TSC diagnosis, the TOSCA PASS did contribute to the mapping of TAND and reproductive clinical features in patients with TSC.

## Data Availability

The raw data supporting the conclusions of this article will be made available by the authors, without undue reservation.
