# Supplementary material for: Real-World Evidence Study on the Long-Term Safety of Everolimus in Patients With Tuberous Sclerosis Complex: Final Analysis Results
Source: Front Pharmacol. 2022 Apr 8;13:802334. doi: 10.3389/fphar.2022.802334 (PMC9023743; doi:10.3389/fphar.2022.802334)
Supplement: Supplementary file 1 [file Table1.DOCX]

**Table S1.** Frequent AEs (>3%, overall), regardless of causality, in the overall population and across age groups

| **Category** | **Overall** | **By age at consent, years** | | | |
| --- | --- | --- | --- | --- | --- |
|  | **N=179** | **≤2**  **N=7** | **>2 to ≤9**  **N=27** | **>9 to <18**  **N=27** | **≥18**  **N=118** |
| **Patients with any frequent AEs, n (%)** | 91 (50.8) | 5 (71.4) | 19 (70.4) | 15 (55.6) | 52 (44.1) |
| Stomatitis | 16 (8.9) | 2 (28.6) | 4 (14.8) | 5 (18.5) | 5 (4.2) |
| Headache | 14 (7.8) | 0 | 1 (3.7) | 3 (11.1) | 10 (8.5) |
| Aphthous ulcer | 13 (7.3) | 1 (14.3) | 4 (14.8) | 1 (3.7) | 7 (5.9) |
| Diarrhea | 13 (7.3) | 1 (14.3) | 4 (14.8) | 1 (3.7) | 7 (5.9) |
| Hypercholesterolemia | 12 (6.7) | 2 (28.6) | 4 (14.8) | 3 (11.1) | 3 (2.5) |
| Vitamin D deficiency | 12 (6.7) | 0 | 0 | 0 | 12 (10.2) |
| Nasopharyngitis | 11 (6.1) | 1 (14.3) | 3 (11.1) | 1 (3.7) | 6 (5.1) |
| Pneumonia | 10 (5.6) | 2 (28.6) | 3 (11.1) | 1 (3.7) | 4 (3.4) |
| Urinary tract infection | 10 (5.6) | 1 (14.3) | 0 | 1 (3.7) | 8 (6.8) |
| Pyrexia | 9 (5.0) | 3 (42.9) | 3 (11.1) | 2 (7.4) | 1 (0.8) |
| Abdominal pain | 8 (4.5) | 0 | 3 (11.1) | 0 | 5 (4.2) |
| Epilepsy | 8 (4.5) | 2 (28.6) | 2 (7.4) | 1 (3.7) | 3 (2.5) |
| Peripheral edema | 8 (4.5) | 0 | 2 (7.4) | 0 | 6 (5.1) |
| Anemia | 7 (3.9) | 0 | 1 (3.7) | 1 (3.7) | 5 (4.2) |
| Bronchitis | 7 (3.9) | 2 (28.6) | 3 (11.1) | 1 (3.7) | 1 (0.8) |
| Hypertension | 7 (3.9) | 0 | 0 | 0 | 7 (5.9) |
| Hypertriglyceridemia | 6 (3.4) | 0 | 0 | 4 (14.8) | 2 (1.7) |
| Influenza | 6 (3.4) | 1 (14.3) | 1 (3.7) | 0 | 4 (3.4) |
| Mouth ulceration | 6 (3.4) | 1 (14.3) | 2 (7.4) | 2 (7.4) | 1 (0.8) |
| Vomiting | 6 (3.4) | 1 (14.3) | 3 (11.1) | 0 | 2 (1.7) |
| **Patients with frequent AEs with CTC grade^a^, n (%)** | 91 (50.8) | 5 (71.4) | 19 (70.4) | 15 (55.6) | 52 (44.1) |
| Grade 1 | 41 (22.9) | 0 | 10 (37.0) | 9 (33.3) | 22 (18.6) |
| Grade 2 | 30 (16.8) | 2 (28.6) | 4 (14.8) | 4 (14.8) | 20 (16.9) |
| Grade 3 | 18 (10.1) | 3 (42.9) | 5 (18.5) | 2 (7.4) | 8 (6.8) |
| Grade 4 | 2 (1.1) | 0 | 0 | 0 | 2 (1.7) |

AEs, adverse events; CTC, Common Terminology Criteria; CTCAE, Common Terminology Criteria for Adverse Events; MedDRA, Medical Dictionary for Regulatory Activities.

MedDRA version 22.1 and CTCAE version 4.03 were used.

^a^If a patient reported multiple frequent AEs, the frequent AE with the worst severity was used.
