# Supplementary material for: Real-World Evidence Study on the Long-Term Safety of Everolimus in Patients With Tuberous Sclerosis Complex: Final Analysis Results
Source: Front Pharmacol. 2022 Apr 8;13:802334. doi: 10.3389/fphar.2022.802334 (PMC9023743; doi:10.3389/fphar.2022.802334)
Supplement: Supplementary file 3 [file Table3.DOCX]

**Table S3.** Reproductive clinical features by age group

| **Category** | **Overall** | **By age at consent, years** | | | |
| --- | --- | --- | --- | --- | --- |
|  | **N=179** | **≤2**  **N=7** | **>2 to ≤9**  **N=27** | **>9 to <18**  **N=27** | **≥18**  **N=118** |
| **Sex** | | | | | |
| Female | 106 (59.2) | 3 (42.9) | 10 (37.0) | 19 (70.4) | 74 (62.7) |
| Male | 73 (40.8) | 4 (57.1) | 17 (63.0) | 8 (29.6) | 44 (37.3) |
| **Patients with abnormal puberty onset** | | | | | |
| Yes^a^ | 4 (2.2) | 0 | 0 | 3 (11.1) | 1 (0.8) |
| Male | 1 (25.0) | 0 | 0 | 1 (33.3) | 0 |
| Female | 3 (75.0) | 0 | 0 | 2 (66.7) | 1 (100.0) |
| No | 160 (89.4) | 0 | 19 (70.4) | 24 (88.9) | 117 (99.2) |
| Not applicable | 15 (8.4) | 7 (100.0) | 8 (29.6) | 0 | 0 |
| **Menstrual cycle disorder (female age ≥10 years)^b^** | 23 (21.7) | 0 | 0 | 5 (26.3) | 18 (24.3) |
| Amenorrhea (female age ≥10 years)^a, b^ | 10 (9.4) | 0 | 0 | 2 (10.5) | 8 (10.8) |
| Amenorrhea lasting >3 months | 6 (60.0) | 0 | 0 | 1 (50.0) | 5 (62.5) |
| **Other abnormal reproductive condition** | 3 (1.7) | 0 | 0 | 2 (7.4) | 1 (0.8) |
| **Hormone tests performed** | 39 (21.8) | 1 (14.3) | 2 (7.4) | 14 (51.9) | 22 (18.6) |
| **Patients with Tanner staging^a^** | 34 (19.0) | 1 (14.3) | 10 (37.0) | 16 (59.3) | 7 (5.9) |
| Male patients with Tanner staging | 6 (17.6) | 0 | 5 (50.0) | 1 (6.3) | 0 |
| Female patients with Tanner staging | 28 (82.4) | 1 (100.0) | 5 (50.0) | 15 (93.8) | 7 (100.0) |
| Male patients with genitalia stage^a,c^ | 6 (8.2) | 0 | 5 (29.4) | 1 (12.5) | 0 |
| Stage 1 | 0 | 0 | 0 | 0 | 0 |
| Stage 2 | 0 | 0 | 0 | 0 | 0 |
| Stage 3 | 1 (16.7) | 0 | 1 (20.0) | 0 | 0 |
| Stage 4 | 3 (50.0) | 0 | 3 (60.0) | 0 | 0 |
| Stage 5 | 2 (33.3) | 0 | 1 (20.0) | 1 (100.0) | 0 |
| Male patients with pubic hair stage^a,c^ | 6 (8.2) | 0 | 5 (29.4) | 1 (12.5) | 0 |
| Stage 1 | 0 | 0 | 0 | 0 | 0 |
| Stage 2 | 0 | 0 | 0 | 0 | 0 |
| Stage 3 | 0 | 0 | 0 | 0 | 0 |
| Stage 4 | 3 (50.0) | 0 | 3 (60.0) | 0 | 0 |
| Stage 5 | 3 (50.0) | 0 | 2 (40.0) | 1 (100.0) | 0 |
| Female patients with breast stage^a,b^ | 25 (23.6) | 0 | 5 (50.0) | 14 (73.7) | 6 (8.1) |
| Stage 1 | 2 (8.0) | 0 | 2 (40.0) | 0 | 0 |
| Stage 2 | 1 (4.0) | 0 | 0 | 1 (7.1) | 0 |
| Stage 3 | 4 (16.0) | 0 | 1 (20.0) | 3 (21.4) | 0 |
| Stage 4 | 2 (8.0) | 0 | 1 (20.0) | 1 (7.1) | 0 |
| Stage 5 | 16 (64.0) | 0 | 1 (20.0) | 9 (64.3) | 6 (100.0) |
| Female patients with pubic hair stage^a,b^ | 26 (24.5) | 0 | 5 (50.0) | 15 (78.9) | 6 (8.1) |
| Stage 1 | 3 (11.5) | 0 | 2 (40.0) | 1 (6.7) | 0 |
| Stage 2 | 1 (3.8) | 0 | 0 | 1 (6.7) | 0 |
| Stage 3 | 3 (11.5) | 0 | 1 (20.0) | 2 (13.3) | 0 |
| Stage 4 | 1 (3.8) | 0 | 0 | 1 (6.7) | 0 |
| Stage 5 | 18 (69.2) | 0 | 2 (40.0) | 10 (66.7) | 6 (100.0) |
| **Patients who used contraception^b^** | 19 (17.9) | 0 | 1 (10.0) | 2 (10.5) | 16 (21.6) |
| **Patients with hormone-based contraception^d^** | 16 (84.2) | 0 | 1 (100.0) | 2 (100.0) | 13 (81.3) |
| Ethinyl estradiol/progestin combination | | | | | |
| Overall^a, e^ | 8 (50.0) | 0 | 0 | 1 (50.0) | 7 (53.8) |
| >50 μg EE | 3 (37.5) | 0 | 0 | 0 | 3 (42.9) |
| <50 μg EE | 5 (62.5) | 0 | 0 | 1 (100.0) | 4 (57.1) |
| Progestin only | | | | | |
| Overall^a, e^ | 8 (50.0) | 0 | 1 (100.0) | 1 (50.0) | 6 (46.2) |
| Pill | 4 (50.0) | 0 | 1 (100.0) | 0 | 3 (50.0) |
| Intrauterine devices | 0 | 0 | 0 | 0 | 0 |
| Depot injection | 3 (37.5) | 0 | 0 | 1 (100.0) | 2 (33.3) |
| Implant | 1 (12.5) | 0 | 0 | 0 | 1 (16.7) |
| **Patients who used external sex hormones^a^** | 5 (2.8) | 0 | 0 | 1 (3.7) | 4 (3.4) |
| Exogenous estrogen | 1 (20.0) | 0 | 0 | 0 | 1 (25.0) |
| Progestin-based to suppress menstrual cycle | 4 (80.0) | 0 | 0 | 1 (100.0) | 3 (75.0) |
| **Patients with ovariectomy** | 3 (1.7) | 0 | 0 | 1 (3.7) | 2 (1.7) |

EE, ethinyl estradiol.

^a^Used as denominator to calculate percent rates for each subcategory. ^b^The percentages use female patients as the denominator. ^c^The percentages use male patients as the denominator. ^d^The percentages use the number of patients with contraception as the denominator. ^e^The percentages use the number of patients with hormone-based contraception as the denominator.

Tanner staging applies as follows: males ≥10 years; females ≥8 years.

Data are represented as n (%).
